# Association of risk factors, clinical presentation, and treatment with neonatal outcomes among pre-eclamptic and eclamptic women: a cross-sectional study

**DOI:** 10.3389/fgwh.2025.1523375

**Published:** 2025-11-11

**Authors:** Syeada Nawal Bukhari, QurratulAin Jamil, Beenish Ihsan, Muhammad Nauman Jamil, Allah Bukhsh, Khezar Hayat, Anees ur Rehman, Jawad Akbar Khan, Shahid Muhammad Iqbal

**Affiliations:** 1 Department of Pharmacy Practice, Faculty of Pharmacy, The Islamia University of Bahawalpur, Bahawalpur, Pakistan; 2 The Children’s Hospital, The University of Child Health Sciences, Lahore, Pakistan; 3 Institute of Pharmaceutical Sciences, University of Veterinary and Animal Sciences, Lahore, Pakistan; 4 Department of Pharmacy Practice, Bahauddin Zakariya University, Multan, Pakistan; 5 Riphah Institute of Pharmaceutical Sciences, Riphah International University, Gulberg Campus, Lahore, Pakistan; 6 Michael Sars Center, University of Bergen, Bergen, Norway

**Keywords:** eclampsia, pre-eclampsia, hypertensive disorders of pregnancy, neonatal outcomes, cross-sectional study, Pakistani women

## Abstract

**Background:**

Eclampsia and pre-eclampsia cause high feto-maternal mortality in Pakistan. This study aimed to identify potential risk factors in pregnant women that can lead to the development of eclampsia and pre-eclampsia.

**Methods:**

A cross-sectional study was conducted in the BVH, Bahawalpur (July 2021–December 2022), to record socio-demographic information, risk factors, clinical symptoms, and treatment administered, which was linked with maternal and neonatal outcomes. The Chi-squared test was applied to describe the relation in categorical variables. Odds ratio were calculated by binary logistic regression for normally distributed significant values.

**Results:**

A total of 85 women with eclampsia and 43 with pre-eclampsia were included in the study. The eclamptic women showed a higher rate of illiteracy, were overweight, and married to their cousins. They presented with high systolic blood pressure, proteinuria, and low platelet count (OR = 7.24, 95% CI = 1.60–32.68, *p* = 0.01), increased respiratory rate, and elevated AST, ALT, and LDH levels at the time of diagnosis. Women administered with MgSO_4_ 10 g/day (48.5% survived vs. 77.4% non-survived) showed high perinatal mortality compared to a 4 g/day dose (30.9% survived vs. 16.1% non-survived) or those who hadn't received magnesium sulfate (20.6% survived vs. 6.5% non-survived).

**Conclusion:**

Advanced maternal age (≥35), overweight, elevated AST, ALT, and LDH levels, consanguinity, and grand multiparity were associated with higher perinatal mortality. Women with these predictive factors should be monitored for the development of pre-eclampsia or eclampsia.

## Highlights

- Eclamptic women showed higher rate of illiteracy, were overweight and in consanguineous relationship
- Clinical presentation included high systolic blood pressure, proteinuria, and low platelet count, tachypnea, and elevated AST, ALT and LDH levels.
- Maternal age ≥35, overweight, grand multiparities and higher dose of MgSO_4_ (10 g/day) were associated with higher perinatal mortality.
- Good antenatal care, early detection, clinical management of disease and better neonatal care can decrease perinatal mortality.

## 1 Introduction

Hypertensive disorders of pregnancy (HDP) are one of the most common causes of maternal and perinatal morbidity and mortality worldwide (1, 2). Around 15–18% of maternal mortalities are directly caused by HDP, including eclampsia and pre-eclampsia (3). HDP are one of the significant hurdles in reducing maternal and perinatal mortalities, hence constituting an obstacle in attaining SDGs (4). Its prevalence varies regionally, with a global average of 116 per 100,000 women, with an annual incidence of approximately 18.1 million HDP cases. In low and middle-income countries (LMICs), there is a significantly high incidence of HDP, resulting in high maternal and neonatal morbidity and mortality compared to high-income countries, 335 vs. 16 per 100,000 live births (5, 6). This global burden is reflected at a national level, where the Pakistan Maternal Mortality Survey 2019 described a maternal mortality rate (MMR) of 186 per 100,000 live births (158 urban vs. 199 rural areas), with HDP accounting for 29% (7).

Pre-eclampsia is characterized by new-onset hypertension (≥140/90 mmHg) in normotensive women with proteinuria (>150 mg protein/day in urine) or significant end-organ dysfunction with or without proteinuria after 20 weeks of pregnancy (8–10). Other clinical symptoms include headache, visual abnormalities, abnormal renal function, chest discomfort, pulmonary edema, poor oxygen saturation, nausea, and abnormal liver enzymes (11). Eclampsia is a serious type of HDP that includes diagnostic features of pre-eclampsia with episodes of tonic-clonic seizures during pregnancy or within 10 days after delivery without other etiologies of seizures (12). In advanced countries, the use of better management strategies for pre-eclampsia resulted in the decreased incidence of eclampsia (1.6–10 cases per 10,000 live births). However, the incidence of both pre-eclampsia and eclampsia is still high in LMICs, standing around 50–151 cases per 10,000 live births, mainly due to poverty, illiteracy, and inaccessible or inadequate healthcare facilities (13, 14). Previously, it was reported that 4.4% of pregnant women were diagnosed with hypertensive disorders of pregnancy in Bahawalpur, Pakistan, and among them, 3.3% had pre-eclampsia and eclampsia (15). Pregnant women with pre-eclampsia and eclampsia may suffer several complications, including renal failure, placental abruption with hemorrhage, liver failure, subcapsular hematoma, HELLP syndrome (hemolysis, elevated liver enzymes, and low platelets), admission to intensive care unit (ICU), and maternal death (16), while oligohydramnios, premature birth, low birth weight, stillbirth, neonatal ICU admission, and even perinatal death are neonatal complications (17, 18).

The Pakistan Maternal Mortality Survey 2019 reported an antenatal care (ANC) coverage of 91% by a skilled person; however, only 52% of women made regular visits. A 32% increase in MMR was observed in 2019 compared to 2017, i.e., 186 vs. 140 per 100,000 live births (7). Only a very few old studies have described the risk factors, clinical symptoms, and maternal and neonatal outcomes of women suffering from pre-eclampsia and eclampsia. In Pakistan, especially Southern Punjab, is an underdeveloped area with limited and overburdened healthcare facilities. Identification of common features, symptoms, and laboratory parameters for pregnant women who are at risk of developing pre-eclampsia and eclampsia can be beneficial for timely diagnosis, resulting in better management with reduced maternal and neonatal outcomes. The present study was designed to identify possible risk factors, clinical presentation, maternal and neonatal outcomes faced by pregnant women suffering from pre-eclampsia and eclampsia.

## 2 Methods

### 2.1 Study design and setting

A cross-sectional study was conducted to examine the association between risk factors, clinical manifestations, treatment, compliance with guidelines, and the prognosis of patients with pre-eclampsia and eclampsia at the Bahawal Victoria Hospital (BVH), Bahawalpur, Pakistan. Study participants were pregnant women (≥18 years) diagnosed with pre-eclampsia or eclampsia and hospitalized in BVH, which is the main tertiary care hospital in Bahawalpur, covering a wide locality of Southern Punjab with 1,600 bed facility. Patients were referred to BVH by a private gynecologist or from Basic Health Units in rural areas. Data for pre-eclampsia was collected from the Department of Obstetrics and Gynecology, BVH, whereas eclamptic patient's data was collected from eclampsia/E-room, BVH. The Department of Obstetrics and Gynecology, BVH, is fully developed with two wards and an outdoor unit, while eclampsia/E-room is established in the Emergency Department for clinical management of eclamptic patients. Specialists from both the Department of Obstetrics and Gynecology and the Department of Emergency are stationed in the eclampsia/E-room for eclamptic patients. A Neonatal follow-up was performed for perinatal outcomes in the Neonatal Intensive Care Unit, Department of Pediatrics and Neonates, BVH, Bahawalpur.

### 2.2 Study population

The inclusion criterion for participants of the study was: i) patients over 18 years old with informed written consent for themselves and their close relatives (husband/parents/siblings/children), ii) patients meeting the diagnostic criteria set out by the International Society for the Study of Hypertension in Pregnancy (ISSHP) (16) iii) patients admitted or referred to BVH. All the patients who were not meeting the inclusion criteria were excluded from the study. All other conditions that can confuse the diagnosis of eclampsia, such as meningitis, hypoglycemia, or alcoholic coma, were also excluded by clinical and laboratory investigation.

In BVH, 167 eclampsia and pre-eclampsia patients were admitted (July 2021–December 2022). The sample size was determined using the online RaoSoft sample size calculator, with a confidence interval of 95% with a 5% margin of error. The calculated sample size was 117, and after adding a 10% margin of incomplete responses, the final sample size was 128.

Informed consent was obtained from patients after informing them regarding the study objectives. They were informed that the collected data would be presented in aggregate without revealing patient identity. If the patient were unable to respond, informed consent was obtained from her first-degree relative or legally authorized representative in the presence of an independent witness. After a thorough literature review and consultation with experts in the field of pharmacy practice and the gynecological department, the data collection tool was developed. Content, construct, and criterion validation of the data collection tool was done by researchers described elsewhere (19). Data was collected between July 2021 to December 2022. Both direct interviews and the patient's medical files were used to collect the data. Patient's socio-demographic information, family, medical history, and pregnancy characteristics, along with blood pressure measurements, were recorded. The laboratory parameters, including complete blood count (CBC), hemoglobin level (Hb), platelet count, serum creatinine level, lactate dehydrogenase (LDH), alanine transaminase (ALT), aspartate transferase (AST), international normalized ratio (INR), and proteinuria, were also obtained. Interviews with participants were conducted to collect any missing/additional information. Participants were asked about symptoms at the time of admission. Clinical management of the patients, including administration of anti-seizure, anti-hypertensive, or any other medications for prevention or palliative care, was recorded. A follow-up was performed to assess the maternal and neonatal outcomes until discharge from the hospital.

### 2.3 Statistical analysis

Data was incorporated into Statistical Package for Social Sciences (IBM Corp, released 2012, IBM SPSS Statistics for Windows, Version 20.0, Armonk, NY: IBM Corp.). Data was summarized using descriptive statistics. Categorical variables were tabulated in numbers (*n*) and percentages, while continuous values were presented as mean ± SEM. Chi-squared test was applied to determine the relation in categorical variables. To determine the link between socio-demographics, clinical features, and treatment with perinatal mortality, odds ratio were calculated by using binary logistic regression of normally distributed significant values. We examined the normality of variables using histograms and the Shapiro–Wilk test, which indicated that the variables were normally distributed, satisfying the normality assumption of binary logistic regression.

### 2.4 Ethical considerations

An approval (Ref no. 144-2022-/PHEC) was obtained from the Pharmacy Human Medical Ethics Committee established by the Islamia University of Bahawalpur. Approval was also taken from the Medical Superintendent, BVH, head of the Department of Obstetrics and Gynecology, and Chief Pharmacist, BVH. The study was conducted according to ethical standards set by the 1964 Helsinki declaration.

## 3 Results

### 3.1 Socio-demographic and patient history

A total of 128 patients who met the inclusion criteria were included in the study. Among them, 85 (66.4%) were suffering from eclampsia, whereas 43 (33.6%) were suffering from pre-eclampsia. Patient's personal and family history show that 54 (63.5%) eclamptic patients were in the age group of 20–34 years, 54 (63.5%) were illiterate, 41 (48.2%) had primiparity, and 58 (68.2%) were married to their first cousins. Eclamptic women presented with comorbidities, including 54 (63.5%) patients who were stressed, 36 (42.4%) were hypertensive, 6 (7.1%) with cardiovascular disorders, and 5 (5.9%) who suffered migraine. The eclamptic women had a family history of hypertension 66 (77.6%), diabetes 43 (50.6%), cardiovascular disorder 35 (41.2%), pre-eclampsia 24 (28.2%), chronic kidney disease 14 (16.5%), epilepsy 4 (4.7%), and thyroid dysfunction 3 (3.5%). On comparison of pre-eclamptic with eclamptic women, illiteracy (*p* = 0.02) and consanguineous union with their first cousins (*p* = 0.01) were linked with the higher likelihood of eclampsia. The eclamptic women also showed a higher tendency of being stressed (63.5% vs. 55.8%), overweight (32.9% vs. 20.9%), cardiovascular disorders (7.1% vs. 2.3%), and migraine (5.9% vs. 2.3%) compared to pre-eclamptic women (Supplementary Table 1).

### 3.2 Clinical and paraclinical characteristics of eclamptic patients

A majority of eclamptic women were having systolic blood pressure in the range of 161–180 mmHg 38 (44.7%), diastolic pressure in the range of 101–120 mmHg 44 (51.8%), and proteinuria in the range of 381–400 mg/dL 30 (38.6%), while platelet count was normal. They were having seizures 2.98 ± 0.13, increased respiratory rate 23.37 ± 1.01, INR 1.10 ± 0.02, creatinine level 1.13 ± 0.15 mg/dL, AST 162.61 ± 0.43 U/L, ALT 39.02 ± 1.37 U/L, and LDH value 477.22 ± 16.96 IU/L. Their common symptoms included headache (91.8%), edema, including both generalized and nondependent (91.8%), epigastric pain (89.4%), and nausea (82.4%). Almost 69.4% of eclamptic women were experiencing blurred vision, 72.9% with tenderness, 36.5% were suffering from pulmonary edema, and 11.8% from hemiplegia. All eclamptic women have experienced grand mal seizures (Supplementary Table 2).

On comparison, eclamptic women had higher systolic (>160 mmHg; 88.2% vs. 37.2%) and diastolic (>100 mmHg; 91.8% vs. 67.4%) blood pressure than pre-eclamptic women. Approximately 36.5% of eclamptic and 21% of pre-eclamptic women had platelet counts ≤1.5 × 10^5^, while respiratory rate, AST, ALT, and LDH were higher in eclamptic women compared to pre-eclamptic women. The eclamptic women experienced epigastric pain (89.4% vs. 65.1%), nausea (82.4% vs. 60.5%), blurred vision (69.4% vs. 48.8%), and pulmonary edema (36.5% vs. 20.9%) more often, compared to pre-eclamptic women (Supplementary Table 2).

### 3.3 Association of demographic and clinical factors with the diagnosis of eclampsia and pre-eclampsia

The association of significant demographic and clinical factors (Table 1), showed eclamptic patients were married to their first cousins, had higher systolic blood pressure (OR is almost doubled on every 20 mmHg increase in blood pressure), proteinuria, platelet count 1.0 × 10^5^–1.5 × 10^5^, increased respiratory rate, elevated liver enzymes, epigastric pain, and nausea.

**Table 1:** Demographic and clinical factors associated with the diagnosis of eclampsia and pre-eclampsia.

Characteristics	Unadjusted odds ratios	Confidence interval 95%	*p*-value
Cousin marriage (Ref: No cousin marriage)	2.71	1.27–5.77	**0**.**01**
Clinical presentation			
Systolic blood pressure (mmHg)			
120–140	Ref	—	—
141–160	1.34	1.08–1.68	**0**.**01**
161–180	3.95	0.94–17.15	0.06
181–200	8.75	1.62–47.0	**0**.**01**
Proteinuria (mg/dL)			
Negative/Trace	Ref	—	**—**
321–340	3.33	0.30–36.11	0.32
341–360	4.87	4.15–230	**0**.**02**
361–380	18.23	4.27–432.68	**0**.**04**
381–400	25.0	1.20–520.73	**0**.**03**
Platelet count/mm^3^			
<1.0 × 10^5^	0.72	0.23–2.16	0.55
1.0 × 10^5^–1.5 × 10^5^	7.24	1.60–32.68	**0**.**01**
>1.5 × 10^5^	Ref	—	—
Respiratory rate	1.06	1.0–1.13	**0**.**04**
AST	1.04	1.0–1.08	**0**.**01**
ALT	1.05	1.01–1.11	**0**.**01**
LDH	1.0	1.0–1.01	**0**.**004**
Epigastric pain	4.52	1.77–11.50	**0**.**002**
Nausea	3.05	1.33–6.98	**0**.**008**

Ref, reference value for comparison.

Association is presented in odds ratio and confidence interval 95% using Binary Logistic Regression.

Statistically significant values are indicated in bold.

### 3.4 Effect of treatment among eclamptic and pre-eclamptic women compared to perinatal outcomes

The majority of eclamptic (89.4%) and pre-eclamptic (69.8%) women received magnesium sulfate (Table 2), followed by labetalol eclamptic (58.8%) compared to pre-eclamptic (62.8%) women, and hydralazine, which was administered in 37.6% eclamptic women compared to 4.7% pre-eclamptic women. Nifedipine and methyl dopa were more frequently administered in pre-eclamptic compared to eclamptic women (81.4% vs. 2.4%) and (53.5% vs. 27.1%) respectively. A significant perinatal mortality was observed in women who received higher doses of magnesium sulfate, i.e., 10 g/day MgSO_4_ (48.5% survived vs. 77.4% not-survived) compared to a 4 g/day dose (30.9% survived vs. 16.1% not-survived). A high perinatal mortality was also observed in patients who were administered hydralazine 5 mg/h, IV (15.5% survived vs. 61.3% not-survived) and nifedipine 40 mg/day, PO (28.9% survived vs. 77.4% not-survived).

**Table 2:** Treatment administered to eclamptic and pre-eclamptic women and fetal outcome.

Treatment	Eclampsia (*n* = 85)	Preeclampsia (*n* = 43)	*p*-value	Feti survived (*n* = 97)	Feti not survived (*n* = 43)	*p*-value	Total (*n* = 128)
Labetolol (IV)							
NA	35 (41.2)	16 (37.2)	0.16	34 (35.1)	17 (54.8)	0.17	51 (39.2)
20 mg/2min	31 (36.5)	23 (53.5)		45 (46.4)	9 (29.0)		54 (42.2)
40 mg/10min	17 (20.0)	4 (9.3)		17 (17.5)	4 (12.9)		21 (16.4)
80 mg/10min	2 (2.4)	0 (0)		1 (1.0)	1 (3.2)		2 (1.6)
Hydralazine (IV infusion)							
NA	53 (62.4)	41 (95.3)	<**0**.**001**	82 (84.5)	12 (38.7)	<**0**.**001**	94 (73.4)
5 mg/h	32 (37.6)	2 (4.7)		15 (15.5)	19 (61.3)		34 (26.6)
Methyldopa-HCl (IV infusion)							
NA	62 (72.9)	20 (46.5)	**0**.**01**	62 (63.9)	20 (64.5)	0.08	82 (64.1)
500 mg/day	13 (15.3)	13 (30.2)		23 (23.7)	3 (9.7)		26 (20.3)
1 g/day	10 (11.8)	10 (23.3)		12 (12.4)	8 (25.8)		20 (15.6)
Nifedipine (PO)							
NA	83 (97.6)	8 (18.6)	<**0**.**001**	69 (71.1)	22 (71.0)	**0**.**03**	91 (71.1)
20 mg/day	2 (2.4)	0 (0)		0 (0)	02 (6.5)		2 (1.6)
40 mg/day	0 (0)	35 (81.4)		28 (28.9)	7 (22.6)		35 (27.3)
Magnesium sulphate (IV infusion)							
NA	9 (10.6)	13 (30.2)	<**0**.**001**	20 (20.6)	2 (6.5)	**0**.**01**	22 (17.2)
4 g/day	8 (9.4)	27 (62.8)		30 (30.9)	5 (16.1)		35 (27.3)
10 g/day	68 (80)	3 (7)		47 (48.5)	24 (77.4)		71 (55.5)

NA, not administered.

Data presented as *n* (%).

Comparison of eclamptic vs. pre-eclamptic women and fetuses survived vs. non-survived using the Chi-squared test.

Statistically significant values are indicated in bold.

### 3.5 Association of socio-demographic and laboratory parameters with the neonatal outcomes

In 85 eclamptic women, 61 neonates survived, whereas in 43 pre-eclamptic women, 36 neonates survived (Table 3). Of the 97 surviving neonates born to both eclamptic and pre-eclamptic women, only 9 (9.3%) neonates born to women >34 years of age survived, while 14 (45.2%) did not survive. Mothers of not-survived fetuses were having >5 parities (32.3% vs. 11.3%), >4 gravida (38.7% vs. 14.4%), and were overweight (51.6% vs. 21.6%) compared to mothers of survived neonates (Table 3). Similarly, mothers of not-surviving fetuses were suffering from increased incidence of seizures (2.74 ± 0.26 vs. 1.86 ± 0.17), having elevated AST value (175.5 ± 5.05 vs. 154.98 ± 2.77), and LDH values (546.53 ± 38.79 vs. 418.34 ± 12.63). The increased odds ratio of non-survival of the fetus was found in mothers aged >34 years, having grand multiparity, and being overweight (Table 4).

**Table 3:** Socio-demographics, clinical and laboratory parameters of women with fetal outcome.

Socio-demographics	Fetus survived (*n* = 97)	Fetus not survived (*n* = 31)	Total (*n* = 128)	F value	*p*-value
Condition^a^					
Eclampsia	61 (62.9)	24 (77.4)	85 (66.4)	—	0.10
Pre-eclampsia	36 (37.1)	7 (22.6)	43 (33.6)		
Age (years)^a^					
<20	16 (16.5)	5 (16.1)	21 (16.4)	—	<**0**.**001**
20–34	72 (74.2)	12 (38.7)	84 (65.6)		
>34	9 (9.3)	14 (45.2)	23 (18.0)		
Parity^a^					
Primiparous	44 (45.4)	14 (45.2)	58 (45.3)	—	**0**.**01**
Multiparous	42 (43.3)	7 (22.6)	49 (38.3)		
Grand multiparous	11 (11.3)	10 (32.3)	21 (16.4)		
BMI^a^					
Underweight (<18.5)	39 (40.2)	9 (29.0)	48 (37.5)	—	**0**.**04**
Normal (18.5–24.9)	37 (38.1)	6 (19.4)	43 (33.6)		
Overweight (25–29.9)	21 (21.6)	16 (51.6)	37 (28.9)		
Cousin marriage^a^					
Yes	59 (60.8)	18 (58.1)	77 (60.2)	—	0.47
No	38 (39.2)	13 (41.9)	51 (39.8)		
Gravida^a^					
1st time	45 (46.4)	14 (45.2)	59 (46.1)	—	**0**.**02**
2nd time	22 (22.7)	2 (6.5)	24 (18.8)		
3rd time	5 (5.2)	1 (3.2)	6 (4.7)		
4th time	11 (11.3)	2 (6.5)	24 (18.8)		
More than 4 times	14 (14.4)	12 (38.7)	26 (20.3)		
Co-morbidities^a^					
(i) Hypertension					
Yes	40 (41.2)	13 (41.9)	53 (41.4)	—	0.55
No	57 (50.8)	18 (58.1)	75 (58.6)		
(ii) Cardiovascular disorders					
Yes	7 (7.2)	0 (0)	7 (5.5)	—	0.12
No	90 (90.2)	31 (100)	121 (94.5)		
(iii) Migraine					
Yes	3 (3.1)	3 (9.7)	6 (4.7)	—	0.15
No	94 (96.9)	28 (90.3)	122 (95.3)		
(iv) Stress					
Yes	57 (58.8)	21 (67.7)	78 (60.9)	—	0.37
No	40 (41.2)	10 (32.3)	50 (39.1)		
Family history^a^					
(i) Diabetes					
Yes	46 (47.4)	20 (64.5)	66 (51.6)	—	0.09
No	51 (52.6)	11 (35.5)	62 (48.4)		
(ii) Hypertension					
Yes	76 (78.4)	27 (87.1)	103 (80.5)	—	0.28
No	21 (21.6)	4 (12.9)	25 (19.5)		
(iii) Cardiovascular disorders					
Yes	40 (41.2)	16 (51.6)	56 (43.8)	—	0.31
No	57 (58.8)	15 (48.4)	72 (56.2)		
(iv) Chronic kidney disease					
Yes	13 (13.4)	10 (32.3)	23 (18)	—	**0**.**01**
No	84 (86.6)	21 (67.7)	105 (82)		
(v) Pre-eclampsia					
Yes	22 (22.7)	14 (45.2)	36 (28.1)	—	**0**.**01**
No	75 (77.3)	17 (54.8)	92 (71.9)		
Clinical investigations of the expecting mother at diagnosis^a^					
(i) Systolic blood pressure (mmHg)					
120–140	8 (8.2)	1 (3.2)	9 (7)	—	0.07
141–160	21 (21.6)	7 (22.6)	28 (21.9)		
161–180	42 (43.3)	8 (25.8)	50 (39.1)		
181–200	22 (22.7)	10 (32.3)	32 (25)		
201–220	4 (4.1)	5 (16.1)	9 (7)		
(ii) Diastolic blood pressure (mmHg)					
80–100	19 (19.6)	2 (6.5)	21 (16.4)	—	0.09
101–120	55 (56.7)	18 (58.1)	73 (57)		
121–140	23 (23.7)	10 (32.3)	33 (25.8)		
141–160	0 (0)	1 (3.2)	1 (0.8)		
(iii) Proteinuria at diagnosis^c^ (mg/dL)					
340–350	6 (13.5)	0 (0)	6 (11.5)	—	**0**.**04**
351–360	8 (18.2)	0 (0)	8 (15.4)		
361–370	11 (25.0)	4 (50.0)	15 (28.8)		
371–380	10 (22.7)	1 (12.5)	11 (21.2)		
381–390	3 (6.8)	3 (37.5)	6 (11.5)		
391–400	6 (13.6)	0 (0)	6 (11.5)		
No of seizures^b^	1.86 ± 0.17	2.74 ± 0.266	2.07 ± 0.148	6.88	**0**.**01**
AST^b^^,^^c^ (U/L)	154.98 ± 2.79	175.50 ± 5.05	157.91 ± 19.98	8.17	**0**.**04**
LDH^b^^,^^d^ (IU/L)	418.34 ± 12.63	546.53 ± 38.79	440.19 ± 13.32	15.28	<**0**.**001**

Statistically significant values are indicated in bold.

a Comparison of mothers of surviving vs. non-survivingfetuses using the Chi-squared test.

b Comparison of mothers of survived and not survived fetuses using One-way ANOVA followed by binary logistic regression on normally distributed data.

c Missing values, *n* = 17.

d Missing values, *n* = 16.

**Table 4:** Association of maternal demographics and clinical parameters with perinatal mortality.

Parameters	Unadjusted odds ratios	Confidence interval 95%	*p*-values
Age (years)			
<20	1.73	0.54–5.54	0.35
20–34	Ref	—	—
>34	8.0	2.84–22.52	<**0**.**001**
Parity			
Primiparous	Ref	—	—
Multiparous	0.52	0.19–1.42	0.20
Grand multiparous	2.87	1.0–8.13	**0**.**04**
BMI			
Underweight (<18.5)	1.42	0.46–4.39	0.53
Normal (18.5–24.9)	Ref	—	—
Overweight (25–29.9)	4.69	1.59–13.84	**0**.**005**
Gravida			
1st time	Ref	—	—
2nd time	0.29	0.06–1.41	0.12
3rd time	0.64	0.06–5.97	0.69
4th time	0.58	0.11–2.95	0.51
More than 4 times	2.75	1.03–7.31	**0**.**04**
Laboratory parameters			
(i) AST	1.02	0.97–1.07	0.32
(ii) LDH	1.0	1.0–1.01	**0**.**001**

Ref, reference value for comparison.

Association is presented in odds ratio and confidence interval 95% using Binary Logistic Regression.

Statistically significant values are indicated in bold.

## 4 Discussion

Pre-eclampsia and eclampsia are serious complications of pregnancy and are on the rise worldwide due to increased BMI and advanced age (20). Social and demographic factors and incomplete maternity care observed in LMICs further augment the risk. In Pakistan, 71% of live births are delivered in a health-care facility, while only 57% women receive complete maternity care (21). We observed a similar kind of behavior where the majority of eclamptic and severe pre-eclamptic women ignored early manifestations of the disease, used home remedies, and obtained medical care when the condition worsened, including having a seizure. There is a consensus on early screening of pre-eclampsia and provision of preventive measures to reduce the MMR. In a populous and resource-limited country like Pakistan, the health-seeking behavior in women depends on several factors, including women's social/financial autonomy and freedom of movement, where approximately 65% women avail healthcare facilities during pregnancy, 43% receive postpartum care, and 39% deliver in the presence of a gynecologist. These factors create a substantial hurdle in achieving a reduction in MMR, which currently stands at 154/100,000 live births (22). In Pakistan, about 57% of maternal deaths are attributed to hemorrhage, pre-eclampsia, and postpartum infections (23).

An increase in systolic blood pressure was associated with an increased occurrence of developing eclampsia. Systolic blood pressure ≥160 mmHg and diastolic blood pressure >110 mmHg are known predictive factors for eclampsia (24, 25). Traditionally, pre-eclampsia is characterized by high blood pressure accompanied by proteinuria in 20-week pregnant women. However, the association of proteinuria with eclampsia/pre-eclampsia is debatable. It is associated with the majority of eclampsia cases, but several researchers also reported cases where proteinuria was absent (26). We observed proteinuria in most patients, with eclamptic women having a higher level of proteinuria as compared to pre-eclamptic women. Although proteinuria can be considered a weak predictor, it can be useful when combined with other indicators in the early diagnosis of women at higher risk (27). In our study, we observed mild thrombocytopenia, which was more frequent in eclamptic (36.5%) compared to pre-eclamptic (21%) women; however, it is not considered a risk factor for severe outcomes (28). The eclamptic and pre-eclamptic women exhibit elevated liver enzymes and LDH levels (29, 30) which we also observed in the present study. Elevated AST and LDH levels were linked to significant outcomes (Table 3). Several studies have reported the association of adverse maternal outcomes with the elevated AST, ALT, LDH, and bilirubin levels, but consensus has not been reached in considering them as predictive factors (31–33). Elevated AST and LDH levels represent multiple organ damage, and if combined with an elevation in bilirubin levels, can indicate hepatic damage also (33, 34). Elevated LDH levels alone can be indicative (34) if associated with elevated liver enzymes and thrombocytopenia, which can prove fatal and require special attention. The respiratory rate was significantly higher in eclamptic women when compared to pre-eclamptic women (*p* = 0.03). This disordered breathing is linked to imbalanced autonomic activity due to high blood pressure (35). In our study majority of eclamptic and pre-eclamptic women complained of epigastric pain (Table 1), which can be considered a predictor of eclampsia along with other factors. We suggest that thrombocytopenia, tachypnoea, elevated ALT, AST, and LDH levels can be used as predictors of severe outcome in eclampsia. Such patients should be monitored regularly, and bilirubin levels should also be checked, which were missing in our study and are considered a limitation of the study.

The majority of the eclamptic and pre-eclamptic women were in the age group of 20–34, which is rational as women are fertile at this age. We observed that first-cousin marriage is a predictor of developing eclampsia in pre-eclamptic women. The effect of consanguinity on the occurrence of pre-eclampsia and eclampsia is debatable. Several studies reported no significant relationship between developing eclampsia and cousin marriages (36–38), while other studies from Asian countries reported an increased risk of developing eclampsia in consanguineous relationships (39, 40). Pakistan has a high consanguinity rate of 59% (36), suggesting further work is required to assess its association with eclampsia.

We compared maternal predictors with neonatal outcomes (Table 3) and observed that 75.78% neonates of pre-eclamptic and eclamptic women survived. The mortality rate of neonates was higher in eclamptic compared to pre-eclamptic women. In this regard, maternal age contributes significantly, as almost half of the neonates who didn't survive were born to mothers >34 years of age (Table 3), which is also reported by other researchers (41, 42). The women suffering from HDPs with ≥5 parity show higher perinatal mortality compared to primiparous or multiparous women. This might be because women having grand multi-parity are also at an advanced age, which acts as a confounding factor (43). The overweight eclamptic or pre-eclamptic women also showed high perinatal mortality. Maternal obesity is associated with a subclinical metabolic inflammatory state and endotoxemia, leading to insulin resistance and mitochondrial dysfunction (44). Other researchers also reported a higher rate of stillbirths in obese women suffering from HDPs (45).

Both antenatal and postnatal care play an important role in reducing maternal and neonatal morbidity and mortality due to HDPs. According to the Pakistan Demographic and Health Survey 2017–2018 (46), 86% of the women received antenatal care (ANC), and among them, younger women were more likely to receive ANC compared to their older counterparts. On the contrary, only 62% women received postnatal care, and this ratio largely remained unchanged since 2012. This was also reflected in our study, where neonatal mortality was high in mothers >34 years of age. So, we strongly suggest improving the quality of ANC and inclusion of aged pregnant women in ANC for early detection of HDPs. Women at higher risk should be counselled properly for lifestyle adjustment during pregnancy, increased monitoring, and provided with postpartum follow-up. The Lady Health Workers should be trained for counselling of pregnant women to avail regular ANC and postnatal care.

### 4.1 Strengths and limitations

The current study is a single-center study. Many patients were admitted by the referral system with missing previous medical records of antenatal care, which were obtained by patient/caretaker or by self-reporting, thus creating a recall bias. Some sociodemographic information, obstetric history/exam, and neonatal health at birth were not assessed. We were also unable to control confounders like age parity, previous gynecological visits, and lab parameters, thus limiting causal inferences. However, in the context of increasing MMR in Pakistan, our study provides workable information and suggests objective measures to improve perinatal outcomes. We recommend that women in consanguineous relationships, of advanced age, and with grand multiparity should be screened for predictive factors of eclampsia/pre-eclampsia and counselled for proper antenatal care. Women at potential risk should be monitored regularly.

## 5 Conclusions

In our study, we evaluated potential risk factors that can lead to the development of eclampsia and pre-eclampsia with associated feto-maternal outcomes in a setting of LMIC like Pakistan. We conclude that early risk identification plays a vital role in reducing maternal and neonatal mortality, which includes the detection of proteinuria and blood pressure monitoring. Women with advanced age (≥35), obese, with grand multiparity, were linked with higher perinatal mortality. Elevated ALT, AST, LDH, bilirubin, thrombocytopenia, and tachypnoea create an augmented risk for severe outcomes, so such patients should be counselled and monitored regularly.

## Data Availability

The original contributions presented in the study are included in the article/Supplementary Material, further inquiries can be directed to the corresponding author.

## Supplementary material

The Supplementary Materialfor this article can be found online at: https://www.frontiersin.org/articles/10.3389/fgwh.2025.1523375/full#supplementary-material
