# Supplementary material for: Association of risk factors, clinical presentation, and treatment with neonatal outcomes among pre-eclamptic and eclamptic women: a cross-sectional study
Source: Front Glob Womens Health. 2025 Nov 11;6:1523375. doi: 10.3389/fgwh.2025.1523375 (PMC12645388; doi:10.3389/fgwh.2025.1523375)
Supplement: Supplementary file 1 [file Datasheet1.docx]

Supplementary Material

# Supplementary Tables

**Table 1**

Demographics, clinical and family history of eclamptic and pre-eclamptic patients on hospital admission.

| Demographic characteristics | Eclampsia  n = 85 (66.4) | Pre-eclampsia n = 43 (33.6) | Total  n = 128 | p-value |
| --- | --- | --- | --- | --- |
| Age (in years) | | | | |
| <20 | 14 (16.5) | 7 (16.3) | 21 (16.4) | 0.68 |
| 20–34 | 54 (63.5) | 30 (69.8) | 84 (65.6) |  |
| >35 | 17 (20) | 6 (14) | 23 (18) |  |
| Education | | | | |
| Illiterate | 54 (63.5) | 17 (39.5) | 71 (55.5) | **0.02** |
| Primary | 14 (16.5) | 8 (18.6) | 22 (17.2) |  |
| Matriculation | 16 (18.8) | 14 (32.6) | 30 (23.4) |  |
| Intermediate | 1 (1.2) | 2 (4.7) | 3 (2.3) |  |
| Graduate | 0 (0.0) | 2 (4.7) | 2 (1.6) |  |
| Parity | | | | |
| Primiparous | 41 (48.2) | 17 (39.5) | 58 (45.3) | 0.64 |
| Multiparous | 31 (36.5) | 18 (41.9) | 49 (38.3) |  |
| Grand multiparous | 13 (15.3) | 8 (18.6) | 21 (16.4) |  |
| BMI | | | | |
| Underweight (<18.5) | 32 (37.6) | 16 (37.2) | 48 (37.5) | 0.25 |
| Normal (18.5–24.9) | 25 (29.4) | 18 (41.9) | 43 (33.6) |  |
| Overweight (25–29.9) | 28 (32.9) | 9 (20.9) | 37 (28.9) |  |
| First cousin marriage | | | | |
| Yes | 58 (68.2) | 19 (44.2) | 77 (60.2) | **0.01** |
| No | 27 (31.8) | 24 (55.8) | 51 (39.8) |  |
| Co morbidities | | | | |
| i) Hypertension | | | | |
| Yes | 36 (42.4) | 17 (39.5) | 53 (41.4) | 0.85 |
| No | 49 (57.6) | 26 (60.5) | 75 (58.6) |  |
| ii) Cardiovascular disorders | | | | |
| Yes | 6 (7.1) | 1 (2.3) | 7 (5.5) | 0.42 |
| No | 79 (92.9) | 42 (97.7) | 121 (94.5) |  |
| iii) Migraine | | | | |
| Yes | 5 (5.9) | 1 (2.3) | 6 (4.7) | 0.66 |
| No | 80 (94.1) | 42 (97.7) | 122 (95.3) |  |
| iv) Autoimmune disease | | | | |
| Yes | 2 (2.4) | 0 (0.0) | 2 (1.6) | 0.55 |
| No | 83 (97.6) | 43 (100) | 126 (98.4) |  |
| v) Stress | | | | |
| Yes | 54 (63.5) | 24 (55.8) | 78 (60.9) | 0.25 |
| No | 31 (36.5) | 19 (44.2) | 50 (39.1) |  |
| Family history of participants of study | | | | |
| i) Diabetes | | | | |
| Yes | 43 (50.6) | 23 (53.6) | 66 (51.6) | 0.45 |
| No | 42 (49.4) | 20 (46.5) | 62 (48.4) |  |
| ii) Hypertension | | | | |
| Yes | 66 (77.6) | 37 (86.0) | 103 (86) | 0.18 |
| No | 19 (22.4) | 6 (14.0) | 25 (14) |  |
| iii) Cardiovascular disorders | | | | |
| Yes | 35 (41.2) | 21 (48.8) | 56 (43.8) | 0.26 |
| No | 50 (58.8) | 22 (51.2) | 72 (56.2) |  |
| iv) Chronic kidney disease | | | | |
| Yes | 14 (16.5) | 9 (20.9) | 23 (18.0) | 0.34 |
| No | 71 (83.5) | 34 (79.1) | 105 (82.0) |  |
| v) Epilepsy | | | | |
| Yes | 4 (4.7) | 5 (11.6) | 9 (7.0) | 0.14 |
| No | 81 (95.3) | 38 (88.4) | 119 (93) |  |
| vi) Pre-eclampsia | | | | |
| Yes | 24 (28.2) | 12 (27.9) | 36 (28.1) | 0.57 |
| No | 61 (71.8) | 31 (72.1) | 92 (71.9) |  |
| vii) Thyroid dysfuntion | | | | |
| Yes | 3 (3.5) | 1 (2.3) | 4 (3.1) | 0.34 |
| No | 82 (96.5) | 41 (95.3) | 123 (96.1) |  |

Data presented as n (%)

Comparison of eclamptic vs. pre-eclamptic women using Chi-squared test

**Table 2**

Clinical presentation and laboratory parameters at the time of diagnosis for eclamptic and pre-eclamptic patients on hospital admission

| Parameters | Eclampsia  n = 85 | Pre-eclampsia n = 43 | Total  n = 128 | F value | p-value |
| --- | --- | --- | --- | --- | --- |
| Systolic blood pressure (mmHg) | | | | | |
| 120–140 | 4 (4.7) | 5 (11.6) | 9 (7.0) | nd | <**0.001^a^** |
| 141–160 | 6 (4.7) | 22 (51.2) | 28 (21.9) |  |  |
| 161–180 | 38 (44.7) | 12 (27.9) | 50 (39.1) |  |  |
| 181–200 | 28 (32.9) | 4 (9.3) | 32 (25) |  |  |
| 201–220 | 9 (10.6) | 0 (0) | 9 (7.0) |  |  |
| Diastolic blood pressure (mmHg) | | | | | |
| 80–100 | 7 (8.2) | 14 (32.6) | 21 (16.4) | nd | <**0.001^a^** |
| 101–120 | 44 (51.8) | 29 (67.4) | 73 (57.0) |  |  |
| 121–140 | 33 (38.8) | 0 (0) | 33 (25.8) |  |  |
| 141–160 | 1 (1.2) | 0 (0) | 1 (0.8) |  |  |
| Proteinuria (mg/dl)^c^ | | | | | |
| Negative/Trace | 11 (14.2) | 7 (17.9) | 18 (15.4) | nd | **0.01^a^** |
| 321–340 | 4 (5.1) | 5 (12.8) | 9 (7.7) |  |  |
| 340–360 | 6 (7.6) | 13 (33.3) | 19 (16.2) |  |  |
| 361–380 | 27 (34.5) | 12 (30.8) | 39 (33.4) |  |  |
| 381–400 | 30 (38.6) | 2 (5.2) | 32 (27.3) |  |  |
| Platelet count/mm^3^ | | | | | |
| <1.0×10^5^ | 8 (9.4) | 7 (16.3) | 15 (11.7) | nd | **0.009^a^** |
| 1.0×10^5^–1.5×10^5^ | 23 (27.1) | 2 (4.7) | 25 (19.5) |  |  |
| >1.5×10^5^ | 54 (63.5) | 34 (79.1) | 88 (68.8) |  |  |
| No. of seizures during disease | 2.98±0.13 | 0.28±0.11 | 2.07±0.14 | 176.16 | <**0.001^b^** |
| Hb (g/dl) | 11.4±0.16 | 11.2±0.29 | 11.33±0.14 | 0.41 | 0.51^b^ |
| Respiratory rate^d^ | 23.37±1.0 | 19.56±1.0 | 22.20±0.83 | 0.45 | **0.03^b^** |
| INR^e^ | 1.10±0.02 | 1.09±0.02 | 1.09±0.01 | 0.00 | 0.98^b^ |
| Creatinine^f^ (mg/dl) | 1.13±0.15 | 1.62±0.35 | 1.29±0.15 | 2.14 | 0.14^b^ |
| AST^g^  (U/L) | 162.61±0.43 | 148±3.01 | 157.91±2.67 | 7.26 | **0.009^b^** |
| ALT^h^ (U/L) | 39.02±1.37 | 33.78±1.57 | 36.79±1.60 | 6.250 | **0.01^b^** |
| LDH^i^ (IU/L) | 477.22±16.96 | 397.85±82.26 | 446.56±12.40 | 10.79 | **0.001^b^** |
| Symptoms at diagnosis | | | | | |
| i) Headache | | | | | |
| Yes | 78 (91.8) | 39 (90.7) | 117 (91.4) | nd | 0.53^a^ |
| No | 7 (8.2) | 4 (9.3) | 11 (8.6) |  |  |
| ii) Blurred Vision | | | | | |
| Yes | 59 (69.4) | 21 (48.8) | 80 (62.5) | nd | 0.19^a^ |
| No | 26 (30.6) | 22 (51.2) | 48 (37.5) |  |  |
| iii) Epigastric pain | | | | | |
| Yes | 76 (89.4) | 28 (65.1) | 104 (81.2) | nd | **0.01^a^** |
| No | 9 (10.6) | 15 (34.9) | 24 (18.8) |  |  |
| iv) Edema | | | | | |
| Yes | 78 (91.8) | 42 (97.7) | 120 (93.6) | nd | 0.18^a^ |
| No | 7 (8.2) | 1 (2.3) | 8 (6.2) |  |  |
| v) Nausea | | | | | |
| Yes | 70 (82.4) | 26 (60.5) | 96 (75) | nd | **0.007^a^** |
| No | 15 (17.6) | 17 (39.5) | 32 (25) |  |  |
| vi) Pulmonary edema | | | | | |
| Yes | 31 (36.5) | 9 (20.9) | 40 (31.2) | nd | 0.05^a^ |
| No | 54 (63.5) | 34 (79.1) | 88 (68.8) |  |  |
| vii) Hemiplegia |  |  |  |  |  |
| Yes | 10 (11.8) | 6 (14) | 16 (12.5) | nd | 0.46^a^ |
| No | 75 (88.2) | 37 (86) | 112 (87.5) |  |  |
| viii) Grand mal seizures | | | | | |
| Yes | 85 (100) | 8 (18.5) | 93 (72.7) | nd | <**0.001^a^** |
| No | 0 (0) | 35 (91.5) | 35 (27.3) |  |  |
| ix) Tenderness | | | | | |
| Yes | 62 (72.9) | 36 (85.7) | 98 (76.6) | nd | 0.12^a^ |
| No | 23 (27.1) | 7 (16.3) | 30 (23.4) |  |  |
| x) Parinatal outcome | | | | | |
| Survived | 61 (71.8) | 36 (83.7) | 97 (75.8) | nd | 0.10^a^ |
| Not survived | 24 (28.2) | 7 (16.3) | 31 (24.2) |  |  |

Data presented as n (%) or Mean ± SEM where nd=not determined

^a^Comparison of eclamptic vs. pre-eclamptic women using Chi-squared test

^b^Comparison of eclamptic vs. pre-eclamptic women using One-way ANOVA where p>0.05 was considered significant

^c^Missing values, n=11

^d^Missing values, n=10

^e^Missing values, n=02

^f^Missing values, n=13

^g^Missing values, n=28

^h^Missing values, n=17

^i^Missing values, n=16
