# Supplementary figures and images for: Association of risk factors, clinical presentation, and treatment with neonatal outcomes among pre-eclamptic and eclamptic women: a cross-sectional study
Source: Front Glob Womens Health. 2025 Nov 11;6:1523375. doi: 10.3389/fgwh.2025.1523375 (PMC12645388; doi:10.3389/fgwh.2025.1523375)

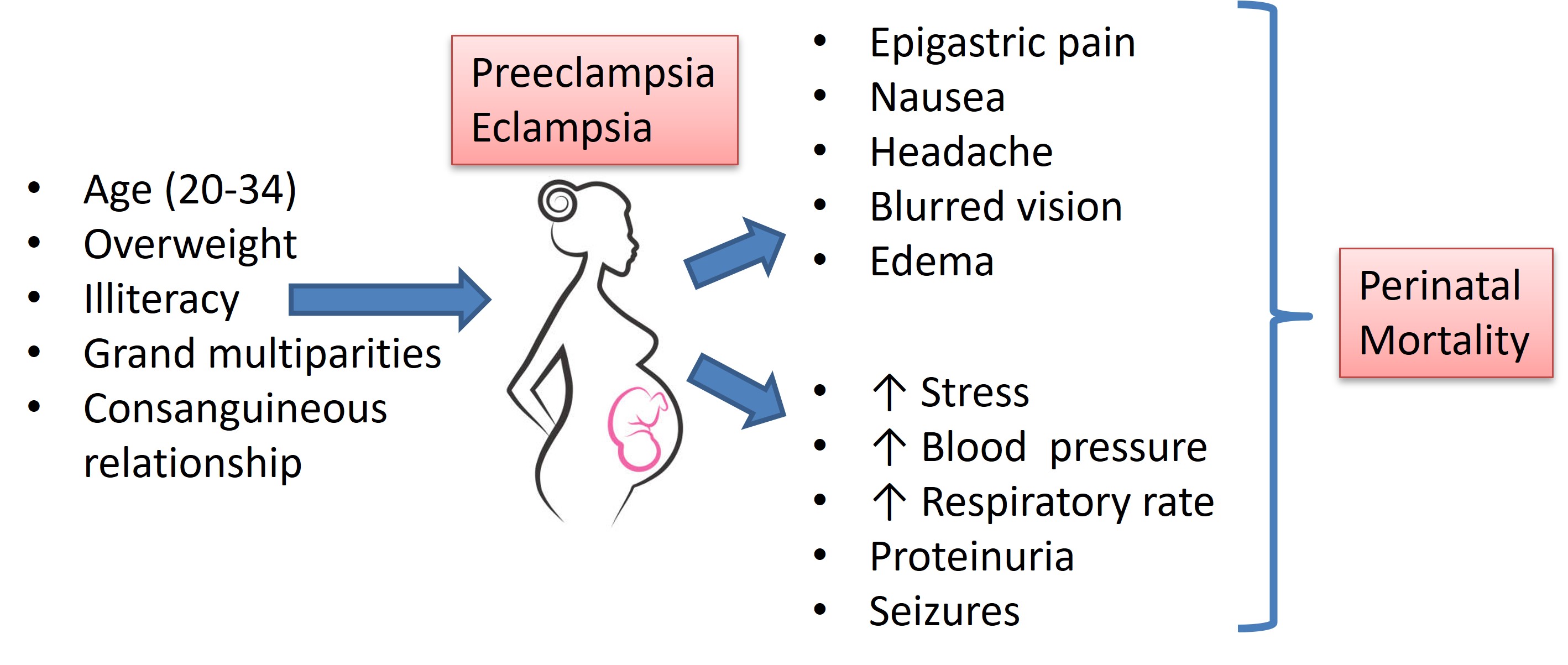

Supplement: Supplementary file 2 [file Image1.jpeg]
